# Coherent photonic Terahertz transmitters compatible with direct comb modulation

**DOI:** 10.1038/s41598-022-13618-y

**Published:** 2022-06-09

**Authors:** Luis Gonzalez-Guerrero, Guillermo Carpintero

**Affiliations:** Grupo de Optoelectronica y Tecnologia Laser (GOTL), Universidad Carlos III de Madrid, 28911 Madrid, Spain

**Keywords:** Fibre optics and optical communications, Optoelectronic devices and components, Electrical and electronic engineering

## Abstract

We present a novel approach to coherent photonic THz systems supporting complex modulation. The proposed scheme uses a single optical path avoiding the problems of current implementations, which include: phase decorrelation, 3-dB power loss, and polarization and power matching circuits. More importantly, we show that our novel approach is compatible with direct modulation of the output of an optical frequency comb (i.e., not requiring the demultiplexing of two tones from the comb), further simplifying the system and enabling an increase in the transmitted RF power for a fixed average optical power injected into the photodiode.

## Introduction

**Figure 1:**
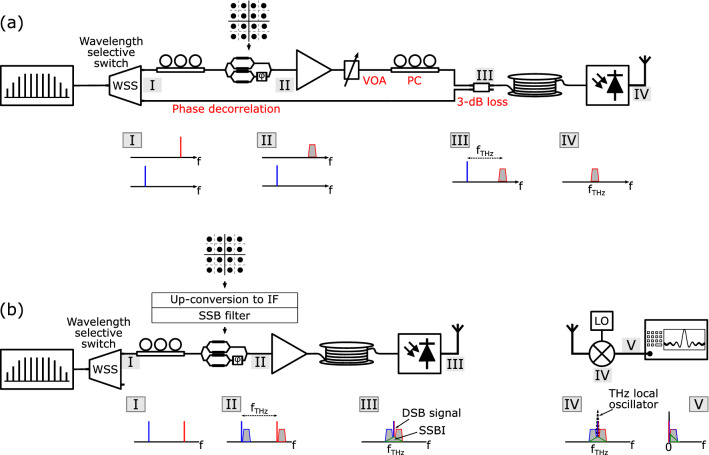
THz transmitters: (**a**) heterodyne transmitter, (**b**) proposed single-path THz transmitter with SSB-C optical modulation and DSB receiver. SSB-C: single sideband with carrier, DSB: double sideband, IF: intermediate frequency, SSBI: signal-signal beat interference.

Terahertz (THz) frequency bands (100 GHz–1 THz) have transmission windows with unregulated widths of several tens of GHz. These bands are considered a key resource to combat the spectrum congestion at lower radio frequencies (RFs). Hence, THz communications have become a very active research topic in the past few years^1^. The highest transmission rates reported in Terahertz (THz) communications have been enabled by coherent photonic systems supporting complex modulation formats^2,3^. To date, the most common architecture for these systems is the one depicted in Fig. 1a. Unlike photonic systems operating at lower mm-wave frequencies—which can generate phase correlated tones with the required frequency spacing by using the suppressed-carrier technique—THz systems rely in an optical frequency comb generator^4^. A wavelength selective switch is then used to demultiplex two tones from the optical frequency comb generator. One of them is encoded with the data and the other is left unmodulated to act as local oscillator in the photomixing process that takes place in the photodiode (PD). Before recombining the two optical modes, it must be ensured that both have the same polarization state and optical power^5^.

The arrangement described before presents several problems for practical implementation: first, as the two comb tones travel through different optical paths, they become decorrelated increasing the phase noise of the generated THz signal. The mitigation of this phenomenon has been the subject of numerous studies, leading to very complex analog^3^ or digital^2^ phase-noise mitigation systems. Second, the system in Fig. 1a leads to a 3-dB optical loss due to need of first splitting the two optical comb modes with the wavelength selective switch and then recombining them with an optical coupler. Furthermore, the need to match both the polarization state (requiring automatic polarization tracking circuits) and the power level (through the use of a variable optical attenuator) of both signals makes the system rather complex.

In this paper, we present a novel approach to coherent photonic THz systems supporting complex modulation that avoids all these problems by using a single optical path as shown in Fig. 1b. Furthermore, since our scheme uses only one port of the wavelength selective switch, it allows a two-fold increase in the number of transmitters. First, we discuss the benefits of using single sideband with carrier (SSB-C) modulation as opposed to double sideband with carrier (DSB-C) modulation for the generation of the optical signal. We then review the different arrangements that can be used to achieve SSB-C modulation and also the techniques available to reduce the amount of distortion at the RF receiver. We run simulations to compare the performance of the proposed single-path THz system to that of the prevailing configuration shown in Fig. 1a. We finalize the first part of the manuscript by discussing the effect of dispersion on the proposed system and the different RF receiver configurations that can be used to downconvert the transmitted THz signal. In the second part of the manuscript, we show how our approach is compatible with the direct modulation of the output of an optical frequency comb generator. This has the potential to further reduce the complexity of the system as the wavelength selective switch is no longer required. Furthermore, we show how this technique can lead to a higher generated THz power for a fixed value of optical power injected into the PD. We finalize the paper by studying the effects of optical dispersion in comb systems and discussing practical implementation issues in the proposed system.

To place this manuscript and its findings within the context of THz photonic generation research, we now enumerate its main contributions:

- This paper proposes, for the first time to the best of the authors knowledge, a THz photonic transmitter that enables the use of a single optical path by simultaneously modulating both optical carriers with the same SSB electrical signal and without the use of any optical filter. The use of single-path photonic mm-wave transmitters has been previously proposed here^6^, however, in such a paper, the authors employ DSB-C modulation plus optical filters to keep only one data-carrying sideband out of the four generated. Using one dedicated optical filter for each transmitter would increase notably the system complexity compared to the architecture proposed in Fig. 1b, where only one filter for N channels (where N is the number of outputs of the wavelength selective switch) is required.
- The most valuable result from the first part of this paper are the simulation results comparing the sensitivity of the proposed system and that of the system in Fig. 1a. Apart from being the first time that both systems are compared, these results, as discussed in the last point of this list, allow us to compute the gain associated to systems based on direct comb modulation.
- The direct modulation of a comb has been demonstrated before^7^. However, the use of SSB modulation and the exploitation of the comb periodicity to achieve generation at higher harmonics is new to this paper. Furthermore, the theoretical study of the dispersion in a modulated comb system is also novel to this manuscript (in reference^7^, the dispersion analysis does not incorporate modulation).
- Finally, the most important contribution of this paper is to derive the power gain achieved with comb systems over the system in Fig. 1a. Up to now, the gain had been calculated for unmodulated comb systems^8,9^ and for generation at the comb frequency repetition. Here we extend these gain calculations to different harmonics of the comb frequency spacing (for both equi-amplitude and Gaussian combs) and use them, in combination with the sensitivity results discussed in the second bullet point, to derive the gain associated to modulated comb systems.

## SSB signal generation and signal-signal beat interference (SSBI) mitigation

The obstacle to using one single optical path in coherent photonic THz transmitters lies in the square-law detection of the PD, which destroys the phase information if both optical tones are modulated with the same baseband complex signal. However, one can turn to a modulation technique commonly used in direct-detection (DD) optical systems to avoid this problem: single sideband with carrier (SSB-C) modulation. As shown in Fig. 1b, by using SSB-C modulation, both optical tones from the comb can be modulated with the same data and still produce a signal after the PD that preserves the phase information. Upon photodetection, the PD generates a double-sideband (DSB) RF signal which can be recovered using a traditional DSB receiver.

**Figure 2:**
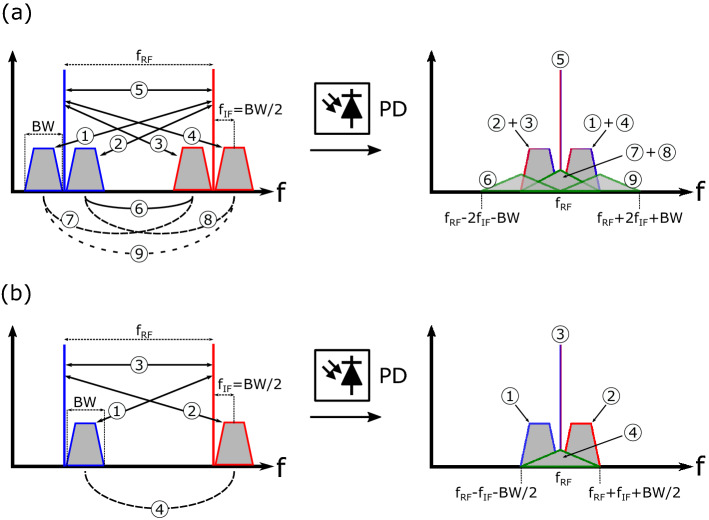
RF beatings generated with (**a**) DSB-C optical modulation, and (**b**) with SSB-C optical modulation.

The phase information can also be preserved if double sideband with carrier (DSB-C) optical modulation is used instead of SSB-C modulation. However, the RF signal generated with this type of modulation contains more signal-signal beat interference (SSBI) terms than those produced with SSB-C modulation. This can be seen in Fig. 2, where all the RF products generated when beating two DSB-C (Fig. 2a) and two SSB-C (Fig. 2b) optical signals are represented. The SSBI terms are represented with green-stroked triangles. As can be seen, the beating between two DSB-C optical signals produces four SSBI products (marked with numbers 6, 9, 7, and 8). These products, apart from distorting the useful signal, also make an inefficient use of the RF spectrum as they stretch outside the bandwidth of the data-carrying signal. Since these SSBI products would be transmitted over the wireless channel, it is crucial to minimize them to comply with the spectrum mask regulations imposed by the wireless telecommunications standards. This can be achieved by using SSB-C optical modulation, which only generates one SSBI product, reducing the amount of distortion and making a more efficient use of the spectrum. For the same data rate, SSB-C modulation halves the occupied RF spectrum and doubles the efficiency of the system.

**Figure 3:**
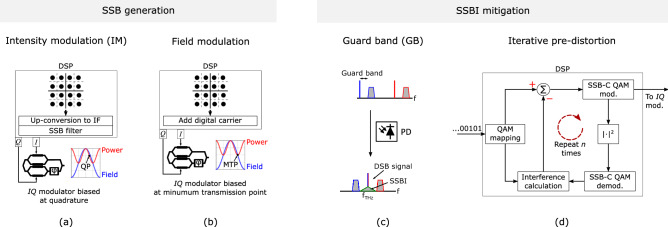
Techniques for the generation of SSB-C signals: (**a**) IM SSB-C, and (**b**) field SSB-C; and techniques for the mitigation of the signal-signal beat interference (SSBI): (**c**) setting a guard band (GB) between carrier and sideband, and (**d**) iterative pre-distortion.

For the generation of the SSB-C signal several techniques can be used. The most common one is the intensity modulation (IM) technique (shown in Fig. 3a), in which the IQ modulator is biased close to the quadrature point to keep a linear relation between the power of the optical signal and the electrical signal driving the modulator. Alternatively, one can bias the modulator in the null point and add the carrier digitally (see Fig. 3b). The advantage of the latter (in this paper referred to as field SSB-C) is that it halves the required digital-to-analog converter bandwidth compared to the IM SSB-C technique^10^. A very important parameter in the SSB-C modulation is the carrier-to-sideband power ratio^11^, CSPR (see equation S13 in the supplementary information for its mathematical definition). In the IM technique, the CSPR is set by fine-tuning the bias point of the optical modulator around the quadrature point. In the field modulation technique, on the other hand, the CSPR is adjusted by simply setting the amplitude of the digital carrier.

Whereas SSB-C optical modulation minimizes the SSBI products compared to DSB-C modulation, it still produces one SSBI product (as shown graphically in Fig. 2b and mathematically in section S1.1 of the supplementary information) that can reduce the sensitivity of the system if no mitigation techniques are employed. A simple way to mitigate this is by allowing a guard band (GB) between optical carrier and data signal. If the GB has the same bandwidth as the data-carrying signal, the SSBI is totally neutralized as shown in Fig. 3c. This technique, however, requires doubling the bandwidth of the digital-to-analog and analog-to-digital converters compared to the case where no GB is used and halves the efficiency of the system. To avoid this, one can use one of the many digital techniques used in DD optical networks to mitigate the SSBI. One that is especially attractive for the system shown in Fig. 1b, is the transmitter-based pre-distortion^12^ (see Fig. 3d). This technique is based on digitally calculating the SSBI and subtracting it from the original signal so that a SSBI-free signal is produced at the PD. The advantage of this technique over receiver-based linearization algorithms is that it mitigates the SSBI before the interference arises, and therefore it minimizes the amount of unwanted signal that gets transmitted over the wireless channel.

## Simulations

**Figure 4:**
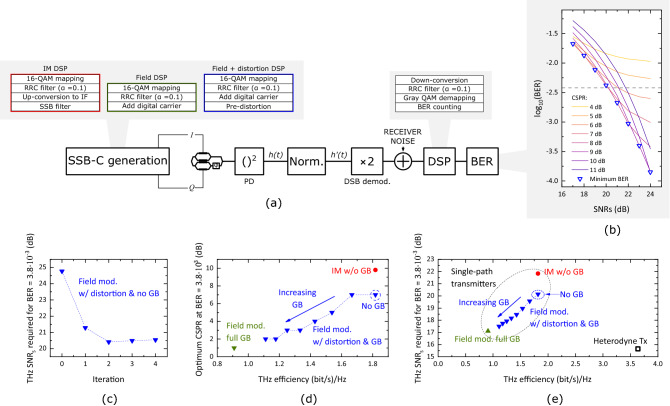
(**a**) Structure of the simulations (*h*(*t*) and $h'(t)$ are mathematically defined in equations S5 and S6 of the supplementary information) and digital signal processing (DSP) used in each SSB-C scheme; (**b**) BER curve obtained for field modulation plus pre-distortion SSBI compensation with no GB; (**c**) sensitivity at a BER of 3.8$\times$10^-3^ versus number of iterations in the pre-distortion technique; (**d**) optimum CSPR versus THz efficiency for each of the SSB-C signals simulated; and (**e**) sensitivity versus THz efficiency of each scheme (including the heterodyne system—black square) at the HD-FEC limit of 3.8$\times$10^-3^.

In order to assess the sensitivity of the single-path photonic THz Tx, transmission simulations were implemented in Matlab using 10-GBd 16-QAM signals and following the structure shown in Fig. 4a. The block with the $\times$2 multiplication accounts for the 3-dB amplitude gain that is achieved with DSB demodulation. The signal-to-noise ratio per symbol ($\hbox {SNR}_{\rm{S}}$) was set by varying the amplitude of receiver noise. Three different SSB-C systems were compared: (a) IM SSB-C with no SSBI compensation, (b) field SSB-C with pre-distortion together with varying widths of GBs, and (c) field SSB-C with a GB as wide as the signal bandwidth. The performance of the heterodyne transmitter depicted in Fig. 1b was also simulated. In such case, differential detection was implemented to account for the phase decorrelation between the two optical paths.

The sensitivity of all the simulated systems was evaluated at the bit error rate (BER) limit of hard decision-forward error correction (HD-FEC), which is 3.8$\times$10^-3^. To find this sensitivty, the BER-versus-$\hbox {SNR}_{\rm{S}}$ curve of each scheme was first calculated by sweeping the amplitude of receiver noise. A straight line was then fitted to the resulting BER-versus-$\hbox {SNR}_{\rm{S}}$ curve by using a y-axis double-log-scale plot. Finally, the intersection between the fitted line and the horizontal line given by $y=\rm{log}_{10}(\rm{log}_{10}(3.8\times 10^{-3}))$ was computed. In the SSB-C schemes, the CSPR was also swept to find the optimum value for each $\hbox {SNR}_{\rm{S}}$ (i.e., that one producing the lowest BER for a given value of $\hbox {SNR}_{\rm{S}}$). An example of one of the generated BER curves is shown in Fig. 4b for the case of field modulation plus pre-distortion SSBI compensation with no GB. The curve used for straight line fitting is the one formed by the blue triangles. The intersection of this curve with a BER of 3.8$\times$10^-3^ corresponds to the “no GB” blue triangle point in Fig. 4e.

Figure 4c shows the sensitivity obtained with the pre-distortion technique against the number of iterations. As two iterations were found to achieve saturated compensation, this was the number of iterations used in the simulations. It is important to mention that the investigation on the minimum required number of iterations was only preformed for the case where no GB was employed. For the cases where a GB is used between carrier and data signal, an even lower number of iterations might suffice. Fig. 4d shows the optimum CSPR for each of the SSB-C signals simulated. As expected, for increasing values of GB, the optimum CSPR value decreases. This is because the SSBI term overlaps less with the useful RF signal and more power can be allocated to the optical data-carrying signal upon SSB-C modulation^11^.

Figure 4e plots the sensitivity of each scheme at the HD-FEC limit against their THz efficiency. As can be seen, the complexity reduction associated with the single-path scheme comes at the expense of lower efficiency and sensitivity compared to the heterodyne approach. The lower efficiency is due to the transmission of a DSB THz signal, whereas the lower sensitivity is explained due to the energy lost in the unmodulated carrier and also the residual SSBI. In spite of this, we note that the simultaneous use of a GB and pre-distortion with the field SSB-C modulation can be a powerful technique to achieve a good trade-off between sensitivity and efficiency in single-path THz transmitters.

## Dispersion considerations and demodulation

**Figure 5:**
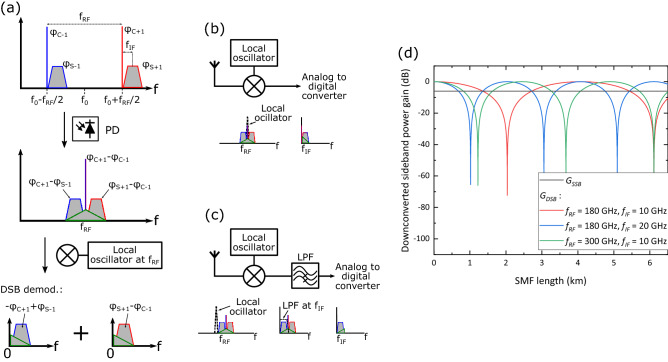
(**a**) Dispersion-induced phase shifts in each of the signals generated in a single-path photonic system with DSB demodulation, $\varphi _{S+1}$, $\varphi _{C+1}$, $\varphi _{S-1}$, and $\varphi _{C-1}$ are the phase shifts due to chromatic dispersion of the carriers and sidebands of the two SSB-C optical signals (all phases are relative to that of the pulse center, which has a frequency of $\hbox {f}_{0}$); (**b**) DSB demodulation receiver; (**c**) SSB demodulation receiver; and (**d**) downconverted sideband power gain versus length of the optical fiber link for the SSB and DSB receivers and various values of $f_{RF}$ and $f_{IF}$ (calculations made with $\beta _{2} = -21.7\,\rm{ps}^{2} /\rm{km}$).

As indicated in Fig. 4a, DSB demodulation was assumed in the simulations. This ensures a 6-dB gain over SSB demodulation but makes the system susceptible to chromatic dispersion effects and, hence, to power-fading. Power fading occurs when two identical signals with a relative phase shift interfere with each other. In the case of the system in Fig. 1b, the process leading to power fading is illustrated in Fig. 5a. As the two SSB-C optical signals travel through a dispersive media such as single-mode fiber (SMF), each of them will experience a different phase shift. This means that the two data-carrying sidebands forming the THz DSB signal will have a relative phase shift. Upon downconversion, these two sidebands will then constructively or destructively interfere with each other depending on this phase shift.

For DSB demodulation, one simply needs a local oscillator with a frequency of $f_{RF}$ as shown in Fig. 5b. In this case, the power gain of the downconverted sideband, $G_{\rm{DSB}}$, varies depending on the length of the optical fiber link according to:

1 $$ G_{{{\text{DSB}}}}  = 0.5 + 0.5\cos (\varphi _{{S + 1}}  - \varphi _{{C - 1}}  + \varphi _{{C + 1}}  - \varphi _{{S - 1}} ) = 0.5 + 0.5\cos \left( {\frac{{  \beta _{2} }}{2}(2\omega _{{RF}} \Omega _{{IF}} )l} \right), $$

where $\varphi _{S+1}$, $\varphi _{C+1}$, $\varphi _{S-1}$, and $\varphi _{C-1}$ are the phase shifts due to chromatic dispersion of the carriers and sidebands as shown in Fig. 5a, $\beta _2$ is the group velocity dispersion of SMF, $\omega _{RF}=2 \pi f_{RF}$, $\Omega _{IF}=2 \pi f_{IF}$, and *l* is the length of SMF. Whereas this type of receiver can yield the highest sideband power, it is sensitive to power fading as shown in Fig. 5d, where the power gain of the downconverted sideband is plotted versus the length of the optical fiber link for both the SSB and DSB receivers and various values of $f_{RF}$ and $f_{IF}$. The gain in the y axis of this figure indicates the gain in sideband power over that used in the simulations, where it was assumed that no relative phase shift existed between the two optical SSB-C signals. As can be seen from Fig. 5d and Eq. ((1)), both $f_{RF}$ and $f_{IF}$ are equally important in determining the interferometric pattern of the DSB receiver.

For SSB demodulation, a local oscillator is also required but with a positive or negative frequency shift with respect to $f_{RF}$ as shown in Fig. 5c. Apart from this, a low pass filter is also needed to filter out the redundant sideband. The gain of the downconverted sideband with this receiver, $G_{\rm{SSB}}$, is

2 $$\begin{aligned} G_{\rm{SSB}} = \frac{1}{4}, \end{aligned}$$

which does not depend on the link length. Thus, this type or receiver is immune to power fading (as shown in Fig. 5d) but it has a 6 dB penalty compared to the maximum gain achievable by the DSB receiver.

It is important to mention that the effect of power fading in the sensitivity of the DSB receiver cannot be calculated by simply subtracting $G_{\rm{DSB}}$ (in dB) to the THz $\hbox {SNR}_{\rm{S}}$ shown in Fig. 4e. This is because the latter takes into account all the power generated at THz frequencies (i.e., including that from the SSBI products), however, $G_{\rm{DSB}}$ refers only to the power of the data-carrying signal and not to that of the SSBI products or the THz carrier. As a final remark, we note that another disadvantage of using optical DSB-C modulation instead of SSB-C modulation is that, even if SSB demodulation is used with the former, the recovered sideband will be subjected to power fading behavior^13^. This is because, as shown in Fig. 2a, each sideband of a THz DSB signal generated with DSB-C modulation is formed by the superposition of two signals.

## Direct comb modulation

**Figure 6:**
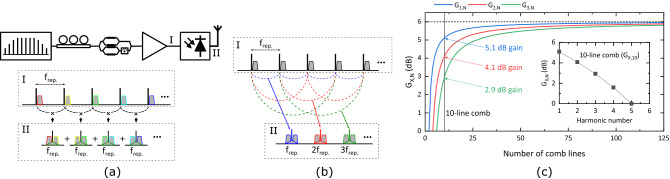
(**a**) Direct-comb-modulation THz transmitter and spectra of the signal before and after the PD, (**b**) signal multiplication in a comb-based THz transmitter, and (**c**) curves for $G_{1,N}$, $G_{2,N}$, and $G_{3,N}$ (inset shows $G_{X,10}$).

The main advantage of using SSB signalling is that is compatible with direct comb modulation as shown in Fig. 6a. This further simplifies the setup in Fig. 1b as not demultiplexing device is needed. Furthermore, if the comb produces transform-limited pulses, this scheme can increase—compared to the two-line modulation described in the previous sections—the emitted THz power for a fixed level of optical power, or photocurrent, in the transmitter PD. A theoretical gain of up to 6 dB was reported here^8^ with experimental results in reference^9^ matching quite well this theoretical value. Calculations in these two references focused on the case where wireless generation is performed at the comb repetition frequency. However, as shown in Fig. 6b, comb modulation allows for RF generation at the harmonics of the comb spacing, relaxing the requirements on the comb repetition frequency. Following the procedure in reference^8^, the gain expression for different harmonics can be derived as (note that the same result is obtained when incorporating SSB-C modulation into the analysis as shown in section S3.1 of the supplementary information):

3 $$\begin{aligned} G_{X,N}=4\bigg (1-\frac{X}{N}\bigg )^2, \end{aligned}$$

where *X* and *N* are the harmonic number and number of comb lines, respectively. It is important to mention that Eq. (3) gives the theoretical value according to ideal square-law detection and perfect phase correlation between comb lines; in practice, the maximum multiplication may be limited by PD saturation effects or phase decorrelation^8^. In Fig. 6c, the curves for $G_{1,N}$, $G_{2,N}$, and $G_{3,N}$ are shown, highlighting the respective gain for a 10-line comb. As could be expected, for a finite number of lines, the gain decreases for higher harmonics. However, all curves approach the same value of 6 dB when the number of lines tends to infinite.

Taking as reference the THz $\hbox {SNR}_{\rm{S}}$ of the heterodyne system at a BER of $3.8\times 10^{-3}$, one can calculate, using Eq. (3), the gain required by a comb system to achieve this BER level. As Eq. (3) is derived assuming a fixed level of average photocurrent, $G_{X,N}$ can only be applied to signals that are normalized in terms of photocurrent. For this, the signals in Fig. 4e have to be multiplied by the scaling factor derived in sections S1.3 (for the SSB-C signals) and S2.3 (for the heterodyne signal) of the supplementary information. Once the signals have been normalized, the THz $\hbox {SNR}_{\rm{S}}$ gain associated with the scaling process has to be computed. Then, the total gain required at the *X*th harmonic of the fundamental frequency of a comb with *N* lines, $G_{req.\,X,N}$, is given by the following expression:

4 $$\begin{aligned} SNR_{SSB-C}=SNR_{Het.}\cdot G_{\langle I_{PD}\rangle }\cdot G_{X,N}\cdot G_{req.\,X,N} \Rightarrow G_{req.\,X,N}= \frac{SNR_{SSB-C}}{SNR_{Het.}\cdot G_{\langle I_{PD}\rangle }\cdot G_{X,N}} \end{aligned}$$

where $SNR_{SSB-C}$ and $SNR_{Het.}$ are the $\hbox {SNR}_{\rm{S}}$ shown in Fig. 4d for the heterodyne and SSB-C signals, respectively, and $G_{\langle I_{PD}\rangle }$ is the energy gain after photocurrent normalization.

**Figure 7:**
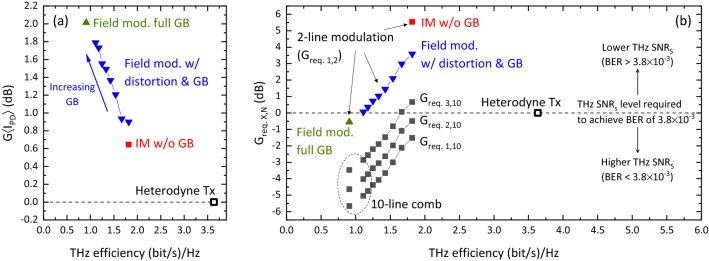
(**a**) $G_{\langle I_{PD}\rangle }$, and (**b**) $G_{req.\,X,N}$ for 2-line modulation (i.e., $G_{req.\,1,2}$) and the modulation of a 10-line comb (i.e., $G_{req.\,X,10}$).

Figure 7a shows $G_{\langle I_{PD}\rangle }$ for each of the SSB-C signals employed in the simulations. The decrease in gain with efficiency is linked to the deviation from a value of 0 dB of the optimum CSPR as the GB is narrowed (see Fig. 4c). Note that, since the energy and average photocurrent normalizations yield the same expression for the heterodyne signal—see sections S2.2 and S2.3 of the supplementary information—its $G_{\langle I_{PD}\rangle }$ has a value of 0 dB. Fig. 7b shows $G_{req.\,X,N}$ for both 2-line modulation (i.e., $G_{req.\,1,2}$) and the modulation of a 10-line comb (i.e., $G_{req.\,X,10}$). As can be seen, even with a modest GB, $G_{req.\,3,10}$ becomes negative, meaning that the $\hbox {SNR}_{\rm{S}}$ achieved in this harmonic is higher than that required for a BER of $3.8\times 10^{-3}$. This increase in link budget may be used to extend the transmission distance of the THz link or achieve a lower BER.

### Gaussian pulses

**Figure 8:**
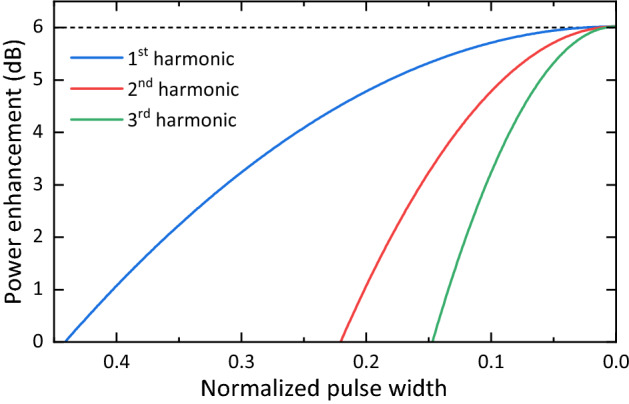
Power gain achieved with a pulsed Gaussian source at different harmonics versus normalized pulse width.

In the previous section, a perfectly flat comb it is assumed. In reality, typical pulsed sources do not produce this type of spectral envelope. One of the most common optical pulses is the Gaussian pulse. The amplitude gain (over two line modulation) achieved at the first harmonic with a pulsed Gaussian source is derived in equation (9) of reference^14^. From this equation, the power gain at different harmonics can be easily derived. This gain is shown in Fig. 8 versus the normalized pulse width for the three first harmonics. The normalized pulse width is the pulse width multiplied by the fundamental repetition frequency. As can be seen, the gain also approaches 6 dB when the normalized pulse width tends to 0 (this limit would correspond to having an infinite number of comb lines). For a known normalized pulse width and harmonic number, the gain required to produce a BER of $3.8\times 10^{-3}$ can be calculated by replacing $G_{X,N}$ in Eq. (4) by the gain shown in Fig. 8.

As an example, let us consider the 60-GHz pulse source (i.e., without the optical clock multiplier) in reference^14^, and assume we want to transmit at 180 GHz (i.e., the third harmonic of 60 GHz). The normalized pulse width of such source is 0.108 (taking the narrowest pulse width value reported in that reference—1,8 ps). The value of the third harmonic curve at this pulse width is approximately 2.8 dB. This value would then be used instead of $G_{X,N}$ in Eq. (4) to find $G_{req.}$. In this case, the SSB-C signal with a THz efficiency of 1.54 (bit/s)/Hz would already have a $\hbox {SNR}_{\rm{S}}$ higher than that required to produce a BER of $3.8\times 10^{-3}$.

## Dispersion in comb-based systems

**Figure 9:**
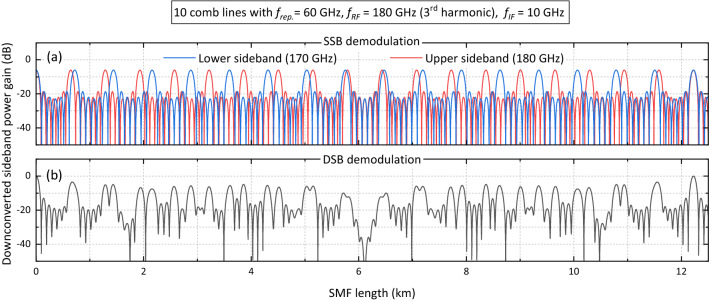
(**a**) Downconverted power gain for the upper and lower sidebands of a 180-GHz DSB-C THz signal generated with an equi-amplitude 10-line comb with a repetition frequency of 60 GHz; (**b**) power gain of the downconverted sideband at $f_{IF}=\rm{10 GHz}$ when DSB demodulation is employed to recover the 180 GHz signal.

Fiber dispersion becomes much more critical in comb-based systems compared to systems based on two-line modulation. Unless a dispersion pre-compensation filter is used, comb-based systems will not be suitable for transmission over long lengths of fiber (this is why the system depicted in Fig. 6a does not contain a fiber spool). Even if SSB demodulation is employed, the recovered signal will be subjected to power fading. To illustrate this, Fig. 9a shows the downconverted power gain for the upper and lower sidebands of a 180-GHz DSB-C THz signal generated with an equi-amplitude 10-line comb with a repetition frequency of 60 GHz (for details on the generation of Fig. 9 see section S3.2 of the supplementary information). The power fading behaviour arises because each component of the DSB-C THz signal is formed by the superposition of many beatings as shown in Fig. 6a. As mentioned before, the maximum power gain achievable with SSB demodulation is 6 dB lower than that obtained with DSB demodulation.

Figure 9b shows the dispersion-induced power gain of the downconverted sideband at $f_{IF}=\rm{10 GHz}$ when DSB demodulation is employed to recover the 180 GHz signal (i.e., using the receiver shown in Fig. 5b). Maximum power is achieved after approximately 12.2 km, which is where the lower and upper sidebands are perfectly in phase as shown in Fig. 9a. Nevertheless, in both schemes, the gain lobe centered at 0 m has a 3-dB bandwidth of several meters that should be enough to implement the system in Fig. 6a for applications where the optical and RF generation units are required to be close to each other.

## Practical implementation issues

Next, we discuss some issues that can arise in the implementation of the systems discussed in this paper:

- In the simulations of Fig. 4, an IQ modulator with an infinite extinction ratio was assumed. The consequence of this is that the suppression of the redundant sideband in the SSB-C modulation is also infinite. In reality, the IQ modulator will have a finite extinction ratio and the unwanted sideband will not be totally suppressed. This can cause interference in systems with dispersion, however, current IQ modulators have extinction ratios of around 30 dB which should be enough to ensure the system SNR is not limited by this.
- In the iterative pre-distortion algorithm implemented in the simulations, the electrical-to-optical block (i.e., the “SSB-C QAM mod.” box in Fig. 3d) was modelled as a linear operator. Because of this, the modulation index (which is defined as the ratio between the peak voltage of the modulating signal and the pi voltage of the modulator) used in the simulations was kept at a relatively low value of 0.25. This was done to avoid hitting the nonlinear region of the IQ modulator. In a practical implementation, a higher modulation index can be used if either the real modulator response is used in the pre-distortion algorithm or a digital algorithm is used to linearize the response of the IQ modulator. This will be particularly important when using higher-order modulation formats such as 64- or 256-QAM.
- In comb-based systems, the maximum multiplication factor will be limited by saturation effects in the PD as discussed in^8^. Hence, it will always be desirable to keep this factor as little as possible by increasing, if possible, the repetition frequency of the comb.
- The fibre connecting the transform-limited pulse source and the PD in Fig. 6a will cause some dispersion in the modes of the transform-limited pulse source. This will lead to a reduction in the gain and also to the introduction of phase noise. The former can be mitigated with dispersion-compensating fibre or programmable filters. The latter may require the use of digital phase noise compensation algorithms, especially when dealing with high-order modulation formats such as 64- or 256-QAM.

## Conclusion

We have presented a photonic THz system compatible with complex modulation that offers several advantages over the typical heterodyne transmitter. By using only one optical path our proposed system solves the most important drawbacks associated with the latter, namely: phase decorrelation of the optical modes, 3-dB power loss, and the necessity for both polarization- and power-matching circuits. Furthermore, as the proposed system uses only one port of the demultiplexing device, it allows a two-fold increase in the number of transmitters. We have also discussed ways to mitigate the SSBI, which arises due to the beating of the two sidebands and can distort the data-carrying signal. We found out that the joint employment of a GB with the pre-distortion technique can achieve a good trade-off between efficiency and sensitivity. Finally, we show that the proposed system is compatible with direct comb modulation. This feature is probably the most important one, not only because it allows a further simplification of the system (no demultiplaxing device is required) but also because it enables an increase in the transmitted THz power for a fixed average optical power injected into the photodiode (PD).

## Supplementary Information

Supplementary Information.
