# Author Correction: Coherent photonic Terahertz transmitters compatible with direct comb modulation

**DOI:** 10.1038/s41598-022-15273-9

**Published:** 2022-07-07

**Authors:** Luis Gonzalez‑Guerrero, Guillermo Carpintero

**Affiliations:** Grupo de Optoelectronica y Tecnologia Laser (GOTL), Universidad Carlos III de Madrid, 28911 Madrid, Spain

Correction to: *Scientific Reports* 10.1038/s41598-022-13618-y, published online 09 June 2022

The original version of this Article contained typographical errors.

Parts of Figure 1, 2, 3 and Figure 5 did not display correctly.

The original Figure 1, 2, 3 and Figure 5 and their accompanying legends appear below.

**Figure 1:**
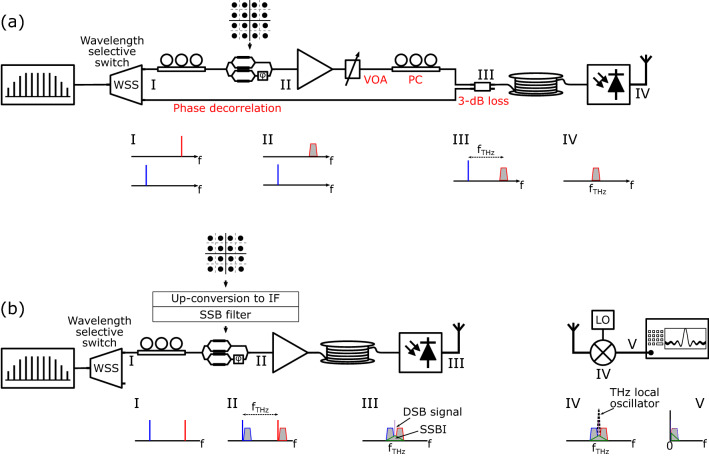
THz transmitters: (**a**) heterodyne transmitter, (**b**) proposed single-path THz transmitter with SSB-C optical modulation and DSB receiver. SSB-C: single sideband with carrier, DSB: double sideband, IF: intermediate frequency, SSBI: signal-signal beat interference.

**Figure 2:**
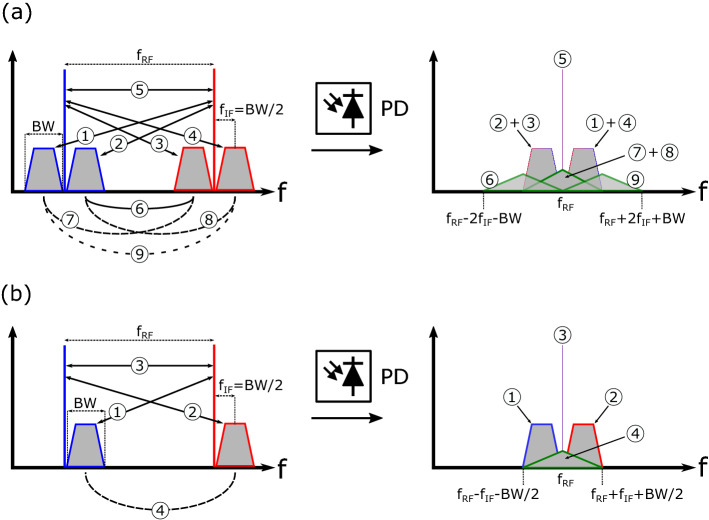
RF beatings generated with (**a**) DSB-C optical modulation, and (**b**) with SSB-C optical modulation.

**Figure 3:**
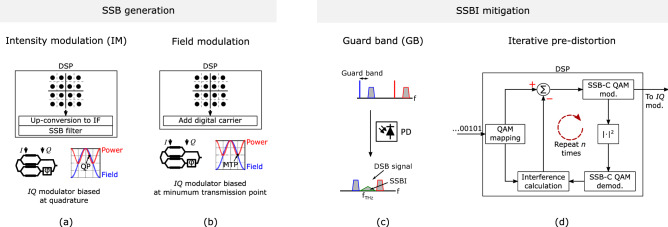
Techniques for the generation of SSB-C signals: (**a**) IM SSB-C, and (**b**) field SSB-C; and techniques for the mitigation of the signal-signal beat interference (SSBI): (**c**) setting a guard band (GB) between carrier and sideband, and (**d**) iterative pre-distortion.

**Figure 5:**
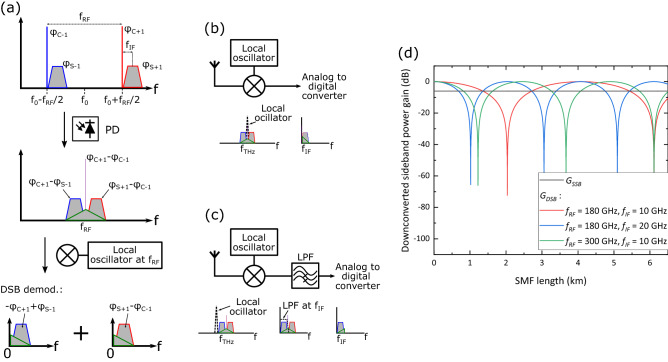
(**a**) Dispersion-induced phase shifts in each of the signals generated in a single-path photonic system with DSB demodulation, $\varphi_{S + 1} ,\,\,\varphi_{C + 1} ,\,\,\varphi_{S - 1} ,\,\,{\text{and}}\,\,\varphi_{C - 1}$ are the phase shifts due to chromatic dispersion of the carriers and sidebands of the two SSB-C optical signals (all phases are relative to that of the pulse center, which has a frequency of f_0_); (**b**) DSB demodulation receiver; (**c**) SSB demodulation receiver; and (**d**) downconverted sideband power gain versus length of the optical fiber link for the SSB and DSB receivers and various values of *f*_RF_ and *f*_IF_ (calculations made with $\beta_{2}$ =  − 21.7 ps^2^/km).

Additionally, equations 1, 18 and 19 contained an error, where “-β2” should read “β2”.

The original Article has been corrected.

