# Supplementary material for: Coherent photonic Terahertz transmitters compatible with direct comb modulation
Source: Sci Rep. 2022 Jun 9;12:9526. doi: 10.1038/s41598-022-13618-y (PMC9184747; doi:10.1038/s41598-022-13618-y)
Supplement: Supplementary file 1 — Supplementary Information. [file 41598_2022_13618_MOESM1_ESM.pdf]

Coherent photonic Terahertz transmitters compatible with direct comb modulation (supplementary information)

Luis Gonzalez-Guerrero[1,*] and Guillermo Carpintero[1]

[1]Grupo de Optoelectronica y Tecnologia Laser (GOTL), Universidad Carlos III de Madrid, Madrid 28911, Spain
[*]lgguerre@ing.uc3m.es

ABSTRACT

We present a novel approach to coherent photonic THz systems supporting complex modulation. The proposed scheme uses a single optical path avoiding the problems of current implementations, which include: phase decorrelation, 3-dB power loss, and polarization and power matching circuits. More importantly, we show that our novel approach is compatible with direct modulation of the output of an optical frequency comb (i.e., not requiring the demultiplexing of two tones from the comb), further simplifying the system and enabling an increase in the transmitted RF power for a fixed average optical power injected into the photodiode (PD).

S1 Two line-modulation single-path transmitter

S1.1 RF generation

As shown in Fig. S1, in the single-path transmitter, two optical carriers, E_1 and E_2, are modulated with the same SSB signal:

$$\begin{aligned} E_1(t) &= A_{SSB}(t)\exp(j\omega_1 t) \\ E_2(t) &= A_{SSB}(t)\exp(j\omega_2 t) \end{aligned} \tag{S1}$$

and

$$A_{SSB}(t) = A_C + A_{mod}(t)\exp(j(\Omega_{IF}t + \theta_{mod}(t))), \tag{S2}$$

where A_C is a constant, $A_{mod}(t)$ and $\theta_{mod}(t)$ are the amplitude and phase of the baseband complex modulation, and Ω_{IF} is an intermediate frequency (IF). When E_1 and E_2 are combined and injected into a PD, the generated photocurrent is proportional to the square modulus of the electric field:

$$I_{PD}(t) \propto |E_1(t) + E_2(t)|^2 = 2|A_{SSB}(t)|^2 + 2|A_{SSB}(t)|^2\cos((\omega_2 - \omega_1)t). \tag{S3}$$

The $I_{PD}(t)$ term at the RF (which is assumed to be in the THz range in the main manuscript) is, thus:

$$I_{RF}(t) \propto 2|A_{SSB}(t)|^2\cos((\omega_2 - \omega_1)t) = \Re\big(2|A_{SSB}(t)|^2\exp(j(\omega_2 - \omega_1)t)\big), \tag{S4}$$

For the Matlab simulations we use the term $2|A_{SSB}(t)|^2$, which is the complex baseband representation of $I_{RF}(t)$. The expansion of this term (which we shall call $h(t)$ hereafter for the sake of brevity) gives:

$$2|A_{SSB}(t)|^2 = h(t) = 2A_C^2 + 2A_{mod}^2(t) + 4A_C A_{mod}(t)\cos(\Omega_{IF}t + \theta_{mod}(t)) = 2A_C^2 + 2A_{mod}^2(t) + h_{mod}(t) \tag{S5}$$

Substituting equation S5 into equation S4, one can see that the $I_{RF}(t)$ signal contains three terms: (a) the data-carrying signal, with an amplitude of $h_{mod}(t)$; (b) the beating of the two carriers, with an amplitude of $2A_C^2$; and (c) the beating of the two sidebands, with an amplitude of $2A_{mod}^2(t)$. Term (c) is the signal-signal beat interference (SSBI) which can distort the useful signal if appropriate mitigation techniques are not employed.

S1.2 RF-energy normalization

To compute the BER curves, the RF signal is normalized in terms of energy, giving:

$$h'(t) = \frac{I_{RF}(t)}{\sqrt{\langle I_{RF}^2(t)\rangle}} = \frac{h(t)}{\sqrt{\langle h^2(t)\rangle}} \tag{S6}$$

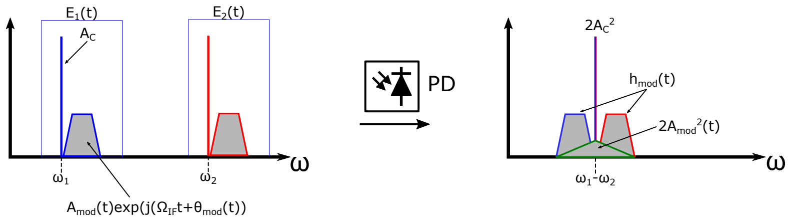

Figure S1. Spectrum before and after photodetection of 2-line SSB-C modulation.

S1.3 Average-photocurrent normalization

For average-photocurrent normalization the RF signal is divided by the average of $I_{PD}(t)$:

$$h'_{DC}(t) = \frac{I_{RF}(t)}{\langle I_{PD}(t)\rangle} = \frac{h(t)}{\langle h(t)\rangle} \tag{S7}$$

S2 Heterodyne transmitter

S2.1 RF generation

In the heterodyne transmitter, only one optical carrier, E_1, is modulated, whereas the other, E_2, is kept unmodulated to act as local oscillator:

$$\begin{aligned} E_1(t) &= A_{mod}(t)\exp(j(\omega_1 t + \theta_{mod}(t))) \\ E_2(t) &= A_C(t)\exp(j\omega_2 t) \end{aligned} \tag{S8}$$

The generated photocurrent in this case is:

$$I_{PD}(t) = A_C^2 + A_{mod}^2(t) + 2A_C A_{mod}(t)\cos((\omega_2-\omega_1)t + \theta_{mod}(t)), \tag{S9}$$

and the RF term is, thus:

$$I_{RF}(t) = 2A_C A_{mod}(t)\cos((\omega_2-\omega_1)t+\theta_{mod}(t)) = \Re\big(A(t)\exp(j\theta_{mod}(t))\exp(j(\omega_2-\omega_1)t)\big), \tag{S10}$$

where $A(t) = 2A_C A_{mod}(t)$. For the Matlab simulations we use the complex baseband representation of $I_{RF}(t)$, which is given by $A(t)\exp(j\theta_{mod}(t))$.

S2.2 RF-energy normalization

For the BER curves, the energy-normalized heterodyne signal is:

$$h'(t) = \frac{I_{RF}(t)}{\sqrt{\langle I_{RF}^2(t)\rangle}} = \frac{A(t)}{\sqrt{\langle A^2(t)\rangle}}\exp(j\theta_{mod}(t)) \tag{S11}$$

S2.3 Average-photocurrent normalization

The normalization of the heterodyne signal in terms of average photocurrent gives:

$$h'_{DC}(t) = \frac{I_{RF}(t)}{\langle I_{PD}(t)\rangle} = \frac{A(t)}{A_C^2 + \langle A_{mod}^2(t)\rangle}\exp(j\theta_{mod}(t)) = \frac{A(t)}{\sqrt{\langle A^2(t)\rangle}}\exp(j\theta_{mod}(t)), \tag{S12}$$

where it is assumed that $A_C^2 = \langle A_{mod}^2(t)\rangle$.

S3 Comb-modulation single-path transmitter

S3.1 Derivation of the gain expression at different harmonics in comb systems with SSB-C modulation

From equation S2, we define the CSPR as:

$$CSPR = \frac{A_C^2}{\langle A_{mod}^2(t)\rangle}. \tag{S13}$$

For N equi-amplitude optical comb lines and an average optical power of one (i.e. $\left\langle |\sum_1^N E_N(t)|^2 \right\rangle = 1$) the power of a single optical carrier (see Fig. S2 for a schematic depiction of the spectrum under consideration) is given by:

$$A_C^2 = \frac{1}{N}\frac{CSPR}{(1+CSPR)} \tag{S14}$$

Following the procedure in reference[1], one can derive the power of the data-carrying term at the first harmonic of the repetition frequency (ω_{rep} in Fig. S2) as:

$$P_{mod\,1,N} = \langle h_{mod\,1,N}^2(t) \rangle = (N-1)^2 2A_C^2 A_{mod}^2 = 2\left(\frac{N-1}{N}\right)^2 \frac{CSPR}{(CSPR+1)^2} \tag{S15}$$

The gain over 2-line modulation at this harmonic is then:

$$G_{1,N} = \frac{P_{mod\,1,N}}{P_{mod\,1,2}} = 4\left(\frac{N-1}{N}\right)^2, \tag{S16}$$

which is the same gain expression obtained in reference[1]. For generation at higher harmonics, the gain expression (i.e., $G_{X,N}$) can be calculated by noticing that the power of the Xth harmonic is given by:

$$P_{mod\,X,N} = (N-X)^2 2A_C^2 A_{mod}^2 \tag{S17}$$

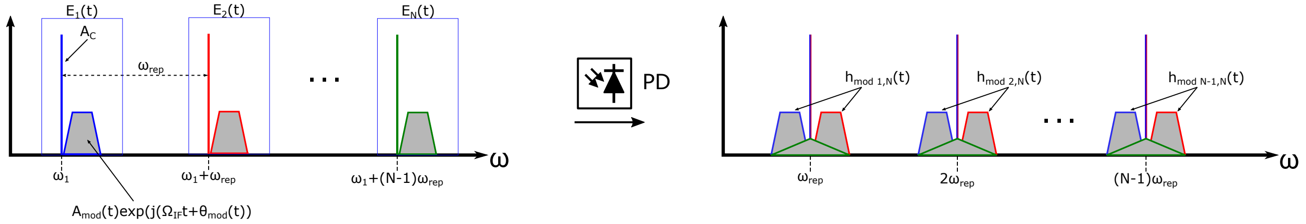

Figure S2. Spectrum before and after photodetection of SSB-C modulation on a comb with N optical lines.

S3.2 Dispersion in comb-based systems

The two sidebands of a THz DSD-C signal (i.e., lower and upper sidebands) generated with an N-line comb can be expressed as a summation of beatings (as shown in Fig. 5 (a) of the main text). If the frequency of the THz signal is equal to the X-th harmonic of the comb repetition frequency, ω_{rep}, then, the electric field of the upper sideband, E_{usb}, can be expressed as:

$$\frac{E_{usb}}{\exp\big(j(X\omega_{rep}+\Omega_{IF})t\big)} = \sum_{n=N_0}^{N_f} A_n^{usb}\exp\Big(j\frac{\beta_2}{2}\big((m^2-n^2)F^2+\Omega_{IF}^2+2mF\Omega_{IF}\big)l\Big) = A_{usb}(l)\exp\big(j\theta_{usb}(l)\big), \tag{S18}$$

where the time-dependent component of the electric field, $\exp\big(j(X\omega_{rep}+\Omega_{IF})t\big)$, has been factored out, A_n^{usb} is the amplitude of each upper sideband beating, and β_2 is the group velocity dispersion of SMF. The rest of the parameters are defined depending on whether the comb has an even or odd number of lines (i.e., whether N is even or odd). For even combs $n = \pm1, \pm3, \pm5...$; $m = n+2X$; $N_0 = -(N-1)$; $N_f = (N-1)-2X$; and $F = \omega_{rep}/2$. For odd combs $n = 0, \pm1, \pm2, \pm3...$; $m = n+X$; $N_0 = -(N-1)/2$; $N_f = (N-1)/2-X$; and $F = \omega_{rep}$. For the lower sideband, the electric field, E_{lsb}, is:

$$\frac{E_{lsb}}{\exp\big(j(X\omega_{rep}-\Omega_{IF})t\big)} = \sum_{n=N_0}^{N_f} A_n^{lsb}\exp\Big(j\frac{\beta_2}{2}\big((m^2-n^2)F^2-\Omega_{IF}^2-2nF\Omega_{IF}\big)l\Big) = A_{lsb}(l)\exp\big(j\theta_{lsb}(l)\big). \tag{S19}$$

For SSB demodulation, the downconverted field, E_{SSB}, is proportional to:

$$\frac{E_{SSB}}{\exp\big(j\Omega_{IF}t\big)} \propto A_{usb/lsb}. \tag{S20}$$

whereas, for DSB demodulation, the downconverted field, E_{DSB}, is:

$$\frac{E_{DSB}}{\exp\big(j\Omega_{IF}t\big)} \propto A_{usb}\exp\big(j\theta_{usb}\big)+A_{lsb}\exp\big(-j\theta_{lsb}\big). \tag{S21}$$

References

1. Kuo, F. M. *et al.* Spectral power enhancement in a 100 GHz photonic millimeter-wave generator enabled by spectral line-by-line pulse shaping. *IEEE Photonics J.* **2**, 719–727, DOI: 10.1109/JPHOT.2010.2064160 (2010).
